# Robust Visuomotor Control for Humanoid Loco-Manipulation Using Hybrid Reinforcement Learning

**DOI:** 10.3390/biomimetics10070469

**Published:** 2025-07-17

**Authors:** Chenzheng Wang, Qiang Huang, Xuechao Chen, Zeyu Zhang, Jing Shi

**Affiliations:** School of Mechatronical Engineering, Beijing Institute of Technology, Beijing 100081, China; czwang@bit.edu.cn (C.W.); chenxuechao@bit.edu.cn (X.C.); 3120245113@bit.edu.cn (Z.Z.); 3220245065@bit.edu.cn (J.S.)

**Keywords:** humanoid robots, humanoid loco-manipulation, intelligent control

## Abstract

Loco-manipulation tasks using humanoid robots have great practical value in various scenarios. While reinforcement learning (RL) has become a powerful tool for versatile and robust whole-body humanoid control, visuomotor control in loco-manipulation tasks with RL remains a great challenge due to their high dimensionality and long-horizon exploration issues. In this paper, we propose a loco-manipulation control framework for humanoid robots that utilizes model-free RL upon model-based control in the robot’s tasks space. It implements a visuomotor policy with depth-image input, and uses mid-way initialization and prioritized experience sampling to accelerate policy convergence. The proposed method is validated on typical loco-manipulation tasks of load carrying and door opening resulting in an overall success rate of 83%, where our framework automatically adjusts the robot motion in reaction to changes in the environment.

## 1. Introduction

In loco-manipulation task, a humanoid robot performs bipedal locomotion and object manipulation simultaneously or alternatively. It involves coordinate movement of all four limbs of a humanoid robot. The ability to perform various loco-manipulation tasks using humanoid robots offers significant benefits to industries that involve logistics, construction, and warehousing. It opens up numerous possibilities for control strategies and robot configurations during a humanoid’s interaction with the environment. For example, humanoid robots have been observed pushing carts with their hands [1], picking and carrying boxes [2] and rolling a large bobbin [3].

Model-based approaches form the foundation for developing humanoid loco-manipulation capabilities. These methods rely heavily on physical models, which play a critical role in shaping the performance, efficiency, and reliability of motion generation and control systems. These methods often structure loco-manipulation tasks as Optimal Control Problems (OCPs), then solving them through either standard or customized numerical solvers. While these optimization-based methods are well-developed, they are not robust against variations in the environment if it cannot be readily modeled with high fidelity.

In recent decade, learning-based methods have been widely applied to robotic control. Among these approaches, Imitation Learning (IL), which involves learning from demonstrations, and Reinforcement Learning (RL) without demonstrations have demonstrated exceptional effectiveness in enabling loco-manipulation tasks. Recent IL advancements have shown promise in scaling to numerous skills [4,5,6] by utilizing extensive demonstration datasets. More demonstration has to be collected for better performance or more versatility, which is not an easy task. RL can overcome this limitation and enable a robot to learn by itself and has successfully orchestrated intricate full-body movements for humanoid robots, such as dance routines [6,7], agile soccer plays [8], and stable locomotion patterns [9,10]. However, RL tends to be excessively inefficient for humanoid loco-manipulation tasks, as they involve high-degree-of-freedom (DoF) robots operating in sparse-reward environments [11]. Consequently, RL-based visuomotor control, which has been well developed in robotic arm manipulation [12,13], remains challenging in humanoid loco-manipulation tasks. Latest work in the field often relies on privileged information [6,14] or human intervention [15,16] to help the robot pick up a box or open a door.

The core of our approach to addressing these challenges involves integrating a wholebody control (WBC) formulation [17] into our policy optimization strategy. Whole-body control serves as a unified control framework that leverages a minimal set of straightforward, low-dimensional rules to fully exploit the capabilities of floating-base robots in achieving compliant, multi-contact interactions with their environment. As opposed to end-to-end methods, our RL policy generates high-level task-space actions, which are subsequently translated into joint-level torque commands for actuation. This not only reduces the degrees of freedom involved in the RL problem, but also addresses the sim-to-real challenge by using WBC to generate safe trajectories. As a loco-manipulation task often involves different control targets at different stages, a multi-stage reward function is introduced to provide a more flexible and denser regard signal, which further increases the training efficiency.

**Figure 1:**
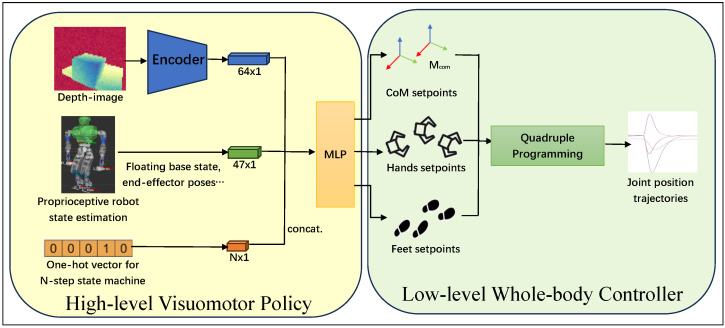
Overall control architecture of our proposed method. The trained policy generates the target task-space command after each motion step, as described in a pre-defined finite state machine, using depth image from the onboard camera in addition to the robot’s proprioceptive feedback. A whole body controller solves for the task-space commands using quadruple programming, computing desired joint positions at 250 Hz, and then sends them to the robot for actuation.

As illustrated in Figure 1, we propose a versatile control method that can cope with various external load for humanoid loco-manipulation tasks with varying environment parameters. Specifically, we build upon our previous work of hybrid humanoid whole-body controlling integrated with reinforcement learning [18] and extend it with depth-imaging and efficient learning techniques. The RL policy is trained in simulation and determines control targets in WBC according to robot state measurements including a depth image of the environment and proprioceptive sensor measurements. The WBC framework generates dynamically feasible and safe robot motion to track the control targets, bridging the sim-to-real gap efficiently. This hybrid structure enables our method to handle various loco-manipulation tasks with different environment parameters.

We study the training efficiency of the proposed control strategy in simulation, where a humanoid robot learns to perform loco-manipulation tasks with varying parameters in a simulated environment. The comparisons show that the techniques proposed by this paper could increase training efficiency by 50–100% and the learned policy could guide the robot to complete different loco-manipulation tasks with more than 90% success rate, whereas an end-to-end controller using PPO [19] does not show any tendency towards convergence. We also deploy the proposed framework to a real-world humanoid robot and achieve an average success rate of 83% in two contact-rich typical loco-manipulation tasks. To the best of our knowledge, this work presents the first successful implementation of deep reinforcement learning of visuomotor policies on loco-manipulation tasks conducted by real-world humanoid systems. The proposed method is developed for general humanoid robot platforms, and it is validated on the 26-DOF BHR10 humanoid robot, whose detailed description could be found in Section 6.1.

To summarize, the principal contributions of this paper are as follows:

- A successful implementation of deep visuo-motor RL for loco-manipulation tasks on a physical humanoid robot, which dynamically adjusts whole-body motions in response to environmental variations such as object displacements.
- A hybrid RL-WBC architecture that bridges the sim-to-real gap by ensuring physical feasibility, achieving high success rates in different loco-manipulation tasks.
- Novel sampling priority metric and mid-way initialization technique that accelerate policy convergence during training.

In the ensuing sections, related works are discussed in Section 2; the visuomotor control framework is explained in Section 3; techniques to increase training efficiency are introduced in Section 4; an ablation study conducted in simulation is presented in Section 5; finally, experiment results of implementing our proposed method on a real humanoid robot are reported in Section 6.

## 2. Related Works

### 2.1. Model-Based Methods

Researchers have applied model-based methods to loco-manipulation task control in humanoid robots, enabling humanoid robots to open a door ([20]), push a cart ([1]), roll a large bobbin ([3]) and etc. These methods are dependent on modeling the physical interaction between the robot and the object to be manipulated, and they are not robust against changes to initial conditions, such as the physics properties of the object and initial displacement of the humanoid robot. For examples, Chappellet et al. [3] apply task-based whole-body control (WBC) and visual SLAM to humanoid robots and successfully enable them to roll heavy and wide bobbin using bimanual grasping and bipedal locomotion at a time. However, this method involves manually pre-defining waypoints for the bobbin’s target pose in the environment for safety concerns. If the bobbin changes either in shape or inertia, the humanoid robot may have to roll it differently, which cannot be robustly solved by the robot itself under this implementation.

### 2.2. Learning-Based Methods

Reinforcement Learning (RL) could help a humanoid robot develop safe behaviors through exploration, especially in locomotion tasks [21,22,23,24,25]. However, RL is subject to the ‘curse of dimensionality’: an increase in problem dimension results in exponential growth in required computational efforts, and humanoid loco-manipulation tasks typically involve over 30 DOFs as opposed to 12–14 DOFs in locomotion tasks alone. Another major challenge in developing RL loco-manipulation task controller is the problem of sparsereward: as opposed to locomotion tasks where similar reward is given at every agent step, in loco-manipulation tasks, rewards are generally only given upon completion of the final goal; collecting it requires many consecutive correct actions from the agent and is very unlikely to happen during exploration. This severely affects the learning efficiency.

Due to the ’curse of dimensionality’, RL controllers designed for robotic locomotion or manipulation tasks only cannot be readily applied; but their handling of the problem of sparse-reward, especially in multi-step manipulation tasks, is still worth discussing. Wang et al. [26] use simple predefined base controllers to bootstrap the target actor network; Nasiriany et al. [13] add an affordance reward term in MAPLE, calculated from the distance between the end-effector and the objects, producing a dense reward signal; and Kuo et al. [27] divides a pick-and-place task into two separate RL problems and train their respective networks separately. The limitation of these methods is that they are only efficient on tasks that have apparent intermediate task goals: such as picking up a certain stale object or leaving it at a location defined by other stale objects. This does not hold true for many loco-manipulation tasks with hard-to-model quantitative dependence among different actions. For example, while pushing a door open, it is difficult to heuristically determine the optimal or suboptimal position and direction of the push motion if the robot stands at different positions and orientations, whose outcome also affects the later movement of walking through the door.

Few RL controllers have been proven effective on full-body control for humanoid robots, and the state-of-the-art is DeepMimic developed by Peng et al. [14], which adds an imitation term to the reward signal, successfully addressing the problem of sparse-reward. However, DeepMimic requires a phase variable to be synchronized with the reference motion clips during imitation learning, limiting the trained policy’s ability to adjust timing of the motion. As a result, this method could only complete tasks with fixed duration or tasks that could be divided into subtasks of fixed durations, which is not always true for loco-manipulation tasks. Additionally, this method has only been validated in simulation.

### 2.3. Hybrid Methods

Both model-based and learning-based methods have their respective advantages and limitations. Model-based methods are more resistant to increase in dimensionality but less robust than learning-based methods. Efforts have been made to incorporate modelbased controllers with learning-based ones so they could complement each other. In the previously mentioned MAPLE controller [13], the lower-layer behavior primitives are implemented as hard-coded closed-loop controllers. And the RL agent’s action vector consists of parameters for these primitives as well as an one-hot vector that selects the proper primitive. This would be less efficient for humanoid robots with four end-effectors as different primitives should be defined for different end-effectors, quadrupling the total number of primitives. Johannink et al. [28] introduces the ResidualRL framework, using RL to generated corrections to base controller outputs and is shown more efficient on a blockassembly task than using RL or a hand-engineered controller alone. However, ResidualRL chooses a parallel structure in which the non-RL and RL parts generate output in the same space. This does not fully utilize the potential of non-RL methods in dimension-reduction thus is unlikely to scale up well to full-body humanoid robots.

The closest to our work is from [12]. They adopts a two-dimensional array to store and improve a state-independent task schema (sequence of existing skills), which selects a preexisting skill to execute at each timestep, and a neural-network to generate state-dependent skill parameters. Their demonstrations are limited to robotic arm tasks with no more than 4 skills and 3 steps. For tasks with longer-horizon and more skills, the task schema array increases rapidly in size and updating it would be very inefficient due to sparse-reward. In contrast, our proposed method implements the state-independent task schema as a heuristically designed FSM. This offloads the learning module from the job of discovering a proper motion sequence, which would be substantially computation-consuming for many long-horizon tasks. It is also easier to combine different skills, such as maintaining balance while reaching for an object, with a predefined task schema. In addition, our proposed method uses WBC to provide skills for humanoid robots with a limited number of parameters.

## 3. Visuomotor Whole Body Control

### 3.1. Whole Body Control with Manipulation Load Stabilizing

Our proposed method uses mc_rtc, an open-source task-oriented robot control framework using WBC ([29]), to generate robot joint trajectories based on control targets such as CoM position or end-effector poses. In this implementation, a floating-based humanoid robot is made of a free-to-move root, *r* revolute joints and r+1 links, which are indexed by k∈{0,…,r}. On the *k*-th link, a set of contact forces $f_{k,1},\ldots ,f_{k,m_{k}}$ are applied at the respective points $a_{k,1},\ldots ,a_{k,m_{k}}$, expressed in the local frame of the *k*-th link. Let $q=\left(x_{0},\theta_{0},\hat{q}\right)\in R^{3+4+r}$ denote the configuration vector of the humanoid robot, where $x_{0}$ is the position of the robot’s root expressed in the global frame, $\theta_{0}$ its orientation as unit quaternion, and $\hat{q}$ the vector of robot actuated joint angles. For each *k*, $J_{tk}\left(p\right)$ denotes the translational Jacobian of the *k*-th link relative to the global frame with respect to q expressed at a local-frame-expressed point p. At each control cycle, the framework computes the joint accelerations that minimize the following cost function:

(1) $$\min_{X}\;f\left(X\right)=\frac{1}{2}X^{T}qX+C^{T}X$$

subject to the following constraints:

(2) $$\begin{array}{cc} M\left(q\right)\ddot{q}+N\left(q,\dot{q}\right)\dot{q}= & M\left(q\right)\left(\;\begin{array}{c} g\\ 0_{4}\\ 0_{r} \end{array}\;\right)+\left(\;\begin{array}{c} 0_{3}\\ 0_{4}\\ u \end{array}\;\right)\\ \; & +\sum_{k,i}J_{tk}{\left(a_{k,i}\right)}^{T}f_{k,i} \end{array}$$

(3) $$\forall k,i\;J_{tk}\left(a_{k,i}\right)\dot{q}=0,$$

(4) $$\forall k,i\;f_{k,i}\in K_{k,i},$$

(5) $$\forall j\;u_{j,\min}\leq u_{j}\leq u_{j,\max}.$$

where g is the vector for gravitational acceleration, u vector for actuation torques at robot joints, $K_{k,i}$ denotes the friction cone at contact point $a_{k,i}$, M(q) is the robot’s inertia matrix of the robot and $N(q,\dot{q})$ the Coriolis and centrifugal force vector. The matrices Q and C are derived from Jacobians between control errors and the robot state. Further explanations of these definitions could be found in the original work of [17].

The calculated joint accelerations are integrated twice to generate joint position commands for the entire robot. More specifically, Equation (1) measures a weighted sum of control error in multiple control objectives, Equation (2) is the equation of motion of the robot system, Equation (3) states that the contact points should not be sliding, Equation (4) states that the friction forces should reside in the corresponding friction cone and Equation (5) states that the control actuation torques should be within the hardware limits of the robot. Exact notation follows Section IV in the original work of [17]. Typical control objectives include end-effector poses, center of mass (COM) position, contact forces and etc. By prioritizing body pose stabilization with the above constraints, the robot maintains overall stability even when minor tracking errors occur due to sim-2-real gap or when the RL policy outputs unsafe movement commands.

Particularly, we extend the humanoid loco-manipulation implementation of [1], whose source code is open. This framework separates external manipulation force errors in the frequency domain through low-pass filtering and apply a ZMP policy to respond to highfrequency errors. However, this approach is affected by the high-frequency noise from the hand force sensors and suffers from phase delay caused by filtering, which contradicts the controller’s requirement for rapid response to high-frequency components. To address this issue, this paper proposes the use of a Generalized Momentum Observer (GMO) to estimate the total external forces acting on the robot. After subtracting the expected foot external forces, the resulting value serves as an estimate of the high-frequency components of the hand contact forces. This improvement induces less phase delay and is not affected by the measurement noise of the force sensors.

### 3.2. Integration with RL

As loco-manipulation tasks often involve a series of different movements, Finite State Machine (FSM) is utilized to define the robot’s whole-body motion for a particular loco-manipulation task, where each FSM state defines one or more control tasks and constraints, enacting a particular movement in the motion sequence. However, when the environment parameters change, this framework cannot decide by itself how to properly adjust the robot movements, or control targets for the contributing control tasks. This is where RL is introduced to increase the robustness of the control architecture.

**Figure 2:**
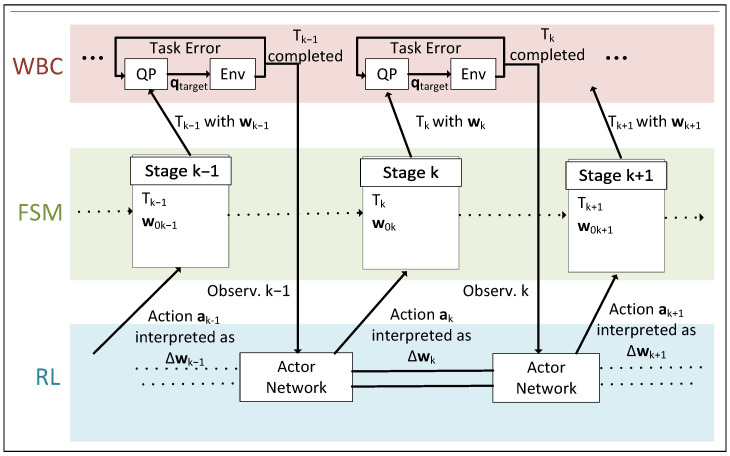
Interactions among WBC, RL and FSM. The FSM is an implementation of the task schema of a long-horizon task. Stage k of the FSM contains task type $T_{k}$ and task parameters $w_{0k}$, which is modified by $\delta w_{k}$ according to action $a_{k}$ generated by the Actor Network of the RL agent, resulting in $w_{k}$. $T_{k}$ and $w_{k}$ are used with WBC to execute the k-th motion in the task schema. Note that the FSM could have branching stages, but this is not illustrated for simplicity purpose.

Our proposed method utilizes the Deep Distributed Distributional Deterministic Policy Gradients (D4PG) RL method [30], to help the robot learn how to make proper adjustments for the next FSM state’s task parameters. The interactions among RL, FSM and WBC are depicted in Figure 2. After one movement step is completed, the immediate reward and observation vector are generated with a unique reward function for each motion step, and the next action is generated from the policy network. Then the next state in the WBC FSM controller is activated, after modifying its control targets based on elements in the action vector. An episode is terminated if the full motion sequence is completed or the robot falls. D4PG is chosen over other common RL algorithms, such as PPO [19], SAC [31] or TD3 [32], because of its efficiency in handling long-term and multi-model rewards, which is characteristic of many loco-manipulation tasks, with N-step return and distributional critic; it also supports learning from certain experiences more frequently by using an experience replay, which will be further explored in Section 4.1.

Formally, our proposed method models each loco-manipulation task as a Markov Decision Process $M=(S,A,P,R,\gamma ,\rho_{0})$, where *S* is the space of states (s∈S), *A* is the space of actions (a∈A), P(·|s,a) is the transition probability, $R(s,a,s^{\prime })$ is the reward function, γ∈[0,1) is the discount factor, and $\rho_{0}\left(\cdot \right)$ is the initial state distribution. Our goal is to learn a closed-loop visuomotor policy $\pi (a_{t}|s_{t})$ that maximizes the expected return $E\left[\sum_{t=0}^{\infty }\gamma^{t}R\left(s_{t},a_{t},s_{t+1}\right)\right]$. In our proposed method, S is the space of the robot’s proprioceptive sensor measurements and depth observation captured by its egocentric camera, A the space of the robot’s commands in the task space of each movement, $R(s,a,s^{\prime })$ the reward function designed for the loco-manipulation task that consists of different terms for different movement steps. More details could be found in our previous work ([18]).

### 3.3. Visuomotor Control with Depth Image

In our previous work [18], the observation vector contains privileged information such as object’s relative displacement to the robot, in addition to proprioceptive robot state information such as estimated values for the robot’s CoM position and velocity, end-effector poses, force sensor readings and etc. To implement visuomotor control, the privileged information in the observation vector could be replaced with a visual-based autoencoder, which is now a common practice in the robotics community [33,34,35,36].

**Figure 3:**
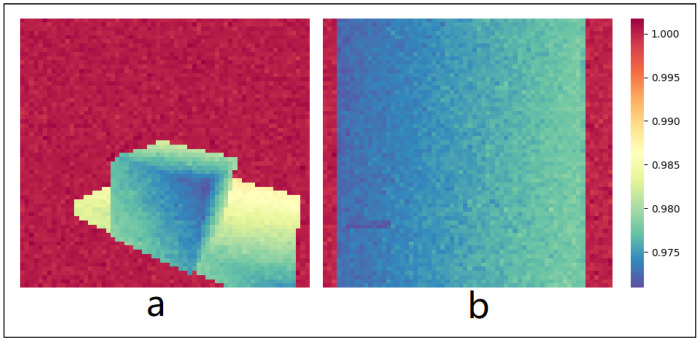
Examples of simulated depth image with noise obtained from the robot egocentric camera in these scenarios: (**a**) load-carrying; (**b**) door-opening. Features of the object and environment are distinctive.

As opposed to using RGB images as input to the autoencoder, we argue that depth images contain sufficient and more direct information for many multi-step loco-manipulation tasks, as they ignore the variable colors in different environments and explicitly show the 3D shape of the objects and their relative displacement to the robot. Depth information has been used in robot control without RGB inputs using both model-based [3,37,38] and learning-based methods [39,40,41]. In our case study in Section 5, the simulated depth images contain distinctive features in both scenarios, as shown in Figure 3.

**Figure 4:**
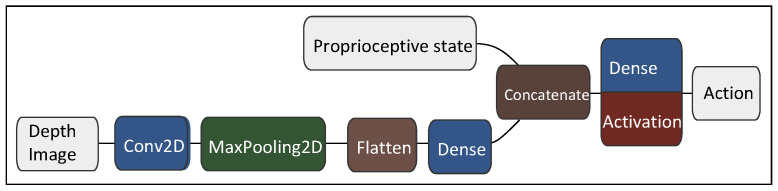
Schematic illustration of the visuo-motor policy network. The 64×64 depth-image is processed by a convolutional layers with 32 8×8 filters and a max-pooling layer, and then processed by 64 fully-connected units after flattening. The resulting latent vector is concatenated with a 47-dimensional proprioceptive state vector, which is obtained from the humanoid’s own state estimation, in addition to a one-hot vector to identify the current FSM state, to form the complete observation vector for the RL agent. It is then fed to a Multilayer Perceptron network with three fully connected layers of dimensions 512,512 and 256, respectively, to generate the action vector. ReLU activation is used in the final layer.

Thus, our proposed method adopts a streamlined imaging process network whose structure is shown in Figure 4. Its output is concatenated with proprioceptive robot state to form the complete observation vector. The visual network is trained together with the policy network and critic network, its weights updated by both critic loss and policy loss.

The imaging network outputs a latent vector to provide more information about the environment in addition to the 6D-pose of the object to be manipulated, which is a common practice in robot manipulation controllers [42]. The novelty in our proposed method is to use only the depth image as input. To verify if the depth image contains the necessary information, we compare in simulation the accuracy in location and orientation estimation of rectangular boxes of different dimensions with our proposed imaging network and several 6D pose estimation frameworks commonly used in robot controlling. The results are displayed in Table 1:

## 4. Training Efficiency Enhancement

### 4.1. Prioritized Experience Sampling

Due to the complexity of loco-manipulation tasks, accomplishing the end goal is uncommon and reward is sparse, which dramatically slows down the training process. One common practice to increase training efficiency for sparse-reward tasks is to use a Prioritized Experience Replay (PER) characterized by a non-uniform sampler, which was first proposed in [43]. Researchers have shown that in general an agent learns faster from experiences that are more successful [44,45,46]. Following this line of thought, we argue that for our proposed control framework, the RL agent would learn faster towards the end goal if it learns more frequently from the experiences where the end goal is met, or almost met.

Thus, we propose a simple metric for prioritizing experience sampling that requires minimal calculation: the priority of sampling a n_step transition from the experience replay is determined by how close the end state is to the terminal state, which marks the accomplishment of the entire task. Formally, when the agent inserts a n-step transition $\left(s,a,r,s^{\prime }\right)$ into the experience, it also registers a priority value *p* given by:

(6) $$p\left(l\right)=\left\{\begin{array}{cc} 1.0, & \mathrm{if}\;l=0\\ 0.3+2*e^{-l}, & \mathrm{otherwise} \end{array}\right.$$

where l is the number of steps between current FSM state and the final state where the end goal is met. As in vanilla D4PG, during training the learner samples transitions from the experience replay to form a batch, and the probability that a transition is sampled is determined by its registered priority.

The standard practice uses TD error as priority metric for the experience replay [43], which has the risk of misleading the agent during exploration to frequently learn from actions that have high intermediate rewards but cannot lead to final completion of the task. In our proposed framework ensures that experiences that end up at or near the end goal will be learned from more frequently, explicitly making the agent learn to complete the entire task, which helps the learner converge to a globally optimal policy for the long-horizon task faster. A comparison between our proposed priorities experience sampling that the standard practice will be provided in Section 5.

### 4.2. Mid-Way Initialization

In reinforcement learning, a common approach for episode initialization is to consistently place the agent in a fixed state. In the task of accomplishing a multi-step loco-manipulation task, a straightforward strategy is to initialize the agent at the first step of the motion sequence, with randomization in some parameters, and let it progress along that sequence throughout the episode. With this design, the policy must sequentially learn all the steps in the motion sequence, beginning with the early steps and gradually advancing to the later ones. However, in many loco-manipulation tasks where midway states are essentially evaluated by whether they could lead to a high-reward terminal state, little progress can be made on the early steps without sufficient visits to the later ones. This poses a self-contradiction. For example, in the task of vehicle ingress, successfully learning to put both feet on the vehicle is essential for the character to achieve a high return from earlier steps such as landing the first foot on the vehicle. If the policy fails to complete the entire ingress task successfully, the earlier steps will result in lower returns, and the agent will not tell whether a landing pose of the first foot is likely to yield high returns in the future.

For multi-step humanoid loco-manipulation tasks, it is often difficult for the robot to reach the later high-reward steps during exploration, as the episodes frequently terminate half-way due to collision or robot falling over. This significantly prohibits effective training. Another drawback of a fixed initial state is the exploration challenge it presents. Until a high-reward state has been visited, the policy has no means of learning that this state is advantageous. In many multi-step loco-manipulation tasks, the terminal state, which marks task completion, is associated with the highest reward and also most difficult to reach. Both of these disadvantages can be mitigated by adjusting the initial state distribution.

For reinforcement learning using human demonstration, the reference trajectory provides a rich and informative state distribution, and the agent could be initialized using samples from the reference trajectory. This strategy has been adopted by previous researchers to facilitate agent training in robotic control using demonstration [14,47,48]. However, our proposed method does not utilize a continuous robot joint reference trajectory. Instead, is has a multi-step motion sequence and a default WBC FSM controller that generates robot trajectories for the default environment parameters. In the motion sequence, some of the midway motion steps start with the robot static and its feet in stable contact with the ground; these steps begin with have dynamically stable robot states, and the robot can be safely initialized at these steps with some randomization.

More specifically, we pick the more stable motion steps and record the default robot pose and joint angles at the beginning of these steps, respectively, using the default initial condition; and at the beginning of each episode, the agent randomly initializes at one of these steps with the corresponding joint angles, which have been proven safe, with some random change to its location and orientation. It is noteworthy that the robot may also reach different safe states with different joint angle values at the aforementioned midway steps, as a result of changes in the initial condition of the first step. Randomization only in the robot displacement does not create a complete representation of the robot state distribution at the mid-way steps. Thus, the mid-way initialization is only used at the early stage of exploration to help the agent encounter high-reward states faster. As the training proceeds, the agent is more frequently initialized at the first step of the motion sequence.

The complete form of our complete proposed method, integrated with depth-based observation, mid-way initialization and prioritized experience replay based on FSM, is illustrated in Algorithm 1. It is adopted from the D4PG algorithm [30].
**Algorithm 1** Visuomotor Control for Humanoid Loco-manipulation Tasks**Require:** batch size *M*, trajectory length *N*, number of actors *K*, replay size *R*, exploration       constant ϵ, initial learning rates $\alpha_{0}$ and $\beta_{0}$**Learner**
  1: Initialize visual encoder weights *v*, which is shared by actor and critic networks (θ,w)  2: Initialize the remaining network weights (θ,w) at random  3: Initialize target weights $\left(\theta^{\prime },w^{\prime }\right)\leftarrow \left(\theta ,w\right)$  4: Launch *K* actors and replicate network weights (θ,w) to each actor  5: **for** 
t=1,…,T **do**  6:       Sample *M* transitions $\left(s_{i:i+N},a_{i:i+N-1},r_{i:i+N-1}\right)$ of length *N* from replay with priority $p_{i}$  7:       Construct the target distributions $Y_{i}=\left(\sum_{n=0}^{N-1}\gamma^{n}r_{i+n}\right)+\gamma^{N}Z_{w^{\prime }}\left(s_{i+N},\pi_{\theta^{\prime }}\left(s_{i+N}\right)\right)$  8:       Compute the actor and critic updates         $\delta_{\theta }={\left.\frac{1}{M}\sum_{i}\nabla_{\theta }\pi_{\theta }\left(s_{i}\right)E\left[\nabla_{a}Z_{w}\left(s_{i},a\right)\right]\right|}_{a=\pi_{\theta }\left(s_{i}\right)}$         $\delta_{w}=\frac{1}{M}\sum_{i}\nabla_{w}{\left(Rp_{i}\right)}^{-1}d\left(Y_{i},Z_{w}\left(s_{i},a_{i}\right)\right)$  9:       Update network parameters $\theta \leftarrow \theta +\alpha_{t}\delta_{\theta },w\leftarrow w+\beta_{t}\delta_{w}$10:       If $t=0\;\mathrm{mod}\;t_{\mathrm{target}\;}$, update the target networks $\left(\theta^{\prime },w^{\prime }\right)\leftarrow \left(\theta ,w\right)$11:       If $t=0\;\mathrm{mod}\;t_{\mathrm{actors}\;}$, replicate network weights to the actors12: **end for**13: return policy parameters θ**Actor**  1: **repeat**  2:     **wait until** current movement step finishes  3:     Sample action $a=\pi_{\theta }\left(s\right)+\epsilon N\left(0,1\right)$  4:     Esecute action a, observe reward *r* and state $s^{\prime }$, which includes an one-hot vector       representing current FSM state  5:     Calculate priority *p* using Equation (6)  6:     Store $\left(s,a,r,s^{\prime },p\right)$ in replay  7: **until** Learner finishes


## 5. Ablation Study

In this section, we conduct an ablation study on the training efficiency of our proposed control framework, which is marked as Method 1 for the rest of this section, in two typical humanoid loco-manipulation scenarios with varying initial conditions: load carrying and door opening. The robot platform in use is BHR10, a full-body humanoid robot developed by the Beijing Institute of Technology. It has a total of 26 actuators, with 6 on legs and 7 on arms. More details of the robot platform can be found in Section 6.1.

For the purpose of ablation study, we apply other methods to these studies. These methods include: our proposed method with the our version of PER as introduced in Section 4.1 replaced by a standard PER that uses TD error as priority metric [43], which is marked as Method 2; our proposed method with initialization only at the first step of the motion sequence as opposed to midway-initialization depicted in Section 4.2, which is marked as Method 3; our proposed method minus the RL component, implemented by overwriting the action vector with all zeros, which is marked as Method 4, and it is taken on average over 100 runs with the same randomization of the environment; and finally, the baseline method, an end-to-end controller that directly generates robot joint trajectories given the same observation vector using vanilla PPO [19].

To further compare with some of the state-of-the-art RL architectures in robot control, we include an additional baseline method, ResidualRL [28], using the WBC layer of our proposed method as its base controller. This implementation uses the policy’s action vector to directly modify joint commands output by the WBC motion controller. In the box manipulation task, the reward signal is determined by distance between the robot hands and the box, as well as the box’s distance from the table. In the door opening task, the instant reward is evaluated by opening angle of the door, as well as the robot displacement with respect to the door (to encourage the robot walk through it).

### 5.1. Load-Carrying Task

**Figure 5:**
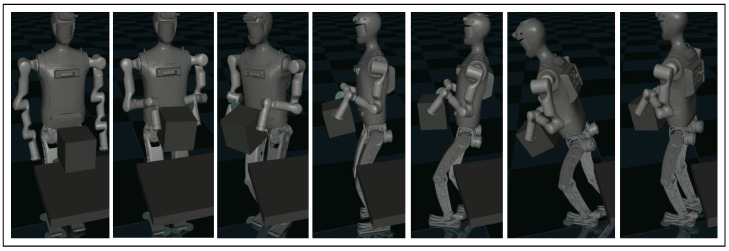
Snapshots of the humanoid robot completing the load-carrying task using our proposed framework.

Load-carrying is a loco-manipulation task that involves the robot picking up a box, turning around and walking away from table with the extra load. The motion sequence involves dozens of steps of different control targets and contact conditions. And there are two more challenges added to this complication: 1. The dimensions, initial pose, and inertial properties of the load could vary; 2. The load is a source of disturbance when the robot maneuvers after picking it up. To address these challenges, in model-based methods, as in [49,50], the physics model of the load is provided. Learning-based methods are efficient in helping a robot to walk steadily with an unknown load, but they need a separate controller to pick up the load from an unknown position. In contrast, our proposed method could help a humanoid robot pick up and carry a box without given information regarding its size, initial pose, or weight. Motion sequence for this task is shown in Figure 5.

In this case study, the initial conditions vary as such: the load’s dimensions randomize in the range of [−0.1 m, +0.1 m] with default value 0.2 m, its mass randomizes in the range of [−2 kg, 2 kg] with default value 3 kg, its position randomizes in the range of [−0.025 m, 0.025 m] in all three directions with default value [0.5 m, 0 m, 0.7 m] and its yaw orientation randomizes in the range of [−0.3 rad, 0.3 rad] with default value 0 rad. The goal of the load-carrying task is to pick up the load and move away from the table so that the robot is prepared to carry the load to other places, which would be a different task.

**Figure 6:**
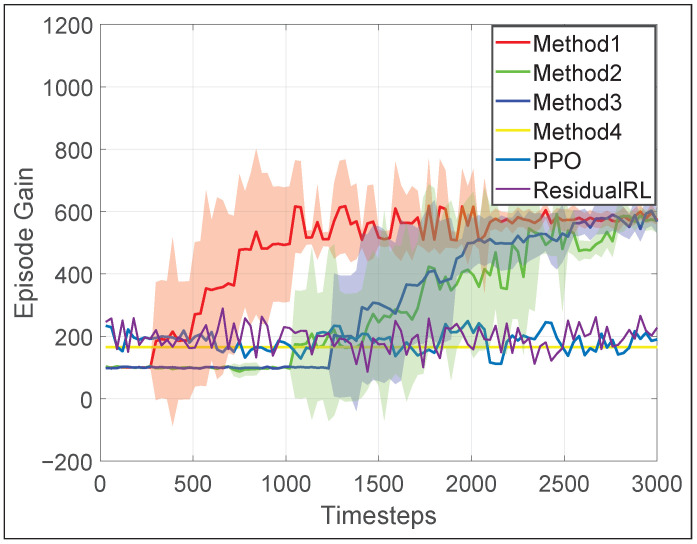
Average episode gain during the training for load-carrying task using different methods (Method 1: our proposed method; Method 2: our method but using standard PER [43]); Method 3: our method minus midway-initialization; Method 4: baseline method without the RL layer).

To observe the improvement of learned policy during the training process, we evaluate the learned policy every 30 learner time steps using the episode gain. Figure 6 presents the average episode gain over the five training sessions throughout the training process, with the shaded area indicating the standard deviation of the episode gain values. Figure 5 contains snapshots of the robot load-carrying motion after 3000 time steps of training using our proposed framework. As shown in Figure 6, Method 1–3 could all converge to a successful jumping motion with in 3000 learner time steps, whereas the end-to-end PPO controller does not display any sign of convergence. Additionally, our proposed framework learns evidently faster, converging in about 2000 time-steps. This shows that for the load-carrying task, either of the training efficiency enhancements introduced in Section 4 could increase training efficiency by 50%. After the policy converges, for Method 1–3, the load-carrying task can be completed with 96% success rate. Neither baseline method shows sign of improvement, mostly likely because it is very difficult for their policy to learn how to determine or adjust each and every robot joints coordinately when the box changes its shape.

### 5.2. Door Opening Task

**Figure 7:**
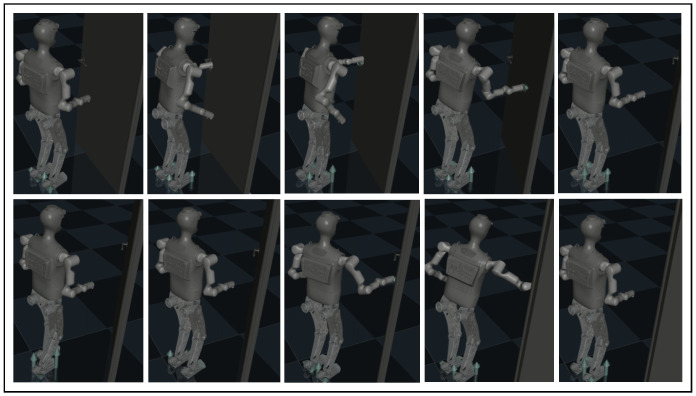
Snapshots of the humanoid robot completing the door-opening task under control of our proposed framework.

The second example of loco-manipulation task control involves door opening. In this scenario, the humanoid robot rotates the door handle to open the door, pushes this door open with both arms alternatively when walking forward intermittently. The motion sequence is shown in Figure 7.

Again, all these steps have different types and values of control targets. The task is also complicated by randomization at initialization: at the beginning of each episode, the humanoid spawns randomly in rectangular area that is 20 cm long and 5 cm wide; it also has a (±15°) random variation in the yaw direction. The training process is repeated four times for Method 1–3.

**Figure 8:**
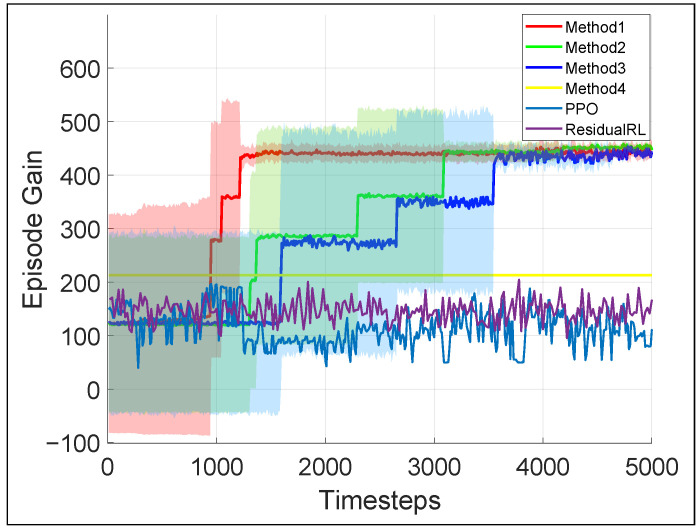
Average episode gain during the training for door opening task for different methods (Method 1: our proposed method; Method 2: our method but using standard PER [43]); Method 3: our method minus midway-initialization; Method 4: baseline method without the RL layer).

Figure 8 presents the average episode gain over the five training sessions throughout the training process of the door-opening task, with the shaded area indicating the standard deviation of the episode gain values. Figure 7 contains snapshots of the robot motion after 5000 learner time steps of training using our proposed framework. As shown by Figure 8, Method 1–3 could all converge to an effective and robust controller for the door-opening task. Evidently, Method 1 has the steepest learning curve and converges in less than 1500 time steps, while it takes Method 2&3 more than 3000 time steps and the end-to-end PPO controller is not converging with this amount of training. This shows that for the door-opening task, either of the training efficiency enhancements introduced in Section 4 could increase training efficiency by more than 100%. After the policy converges, for Method 1–3, the door-opening task can be completed with 92% success rate. Again, the baseline methods display no signs of improvement, mostly likely because it is very difficult for their policy to learn how to determine or adjust all the robot joints coordinately when the robot considerably changes its relative position and orientation to the door.

It is noteworthy that the learning curves for the door-opening task are ‘discontinuous’, which have multiple steep jumps or bumps. This is unlike more RL applications but not surprising as our proposed framework uses a family of reward functions and a large amount of reward could only be obtained when the end goal is accomplished. In fact, in this door-opening task controller, it is unclear how to quantitatively guide the robot’s motion midway, so most of the episode reward is given only when the robot successfully completes the entire task.If the humanoid robot loses balance or fails to unlock the door, the agent loses the large end-goal reward term entirely, thus the discontinuity. With many more repeated training sessions, the average episode gain curve should become smoother.

## 6. Experiment Results

### 6.1. Robot Platform

**Figure 9:**
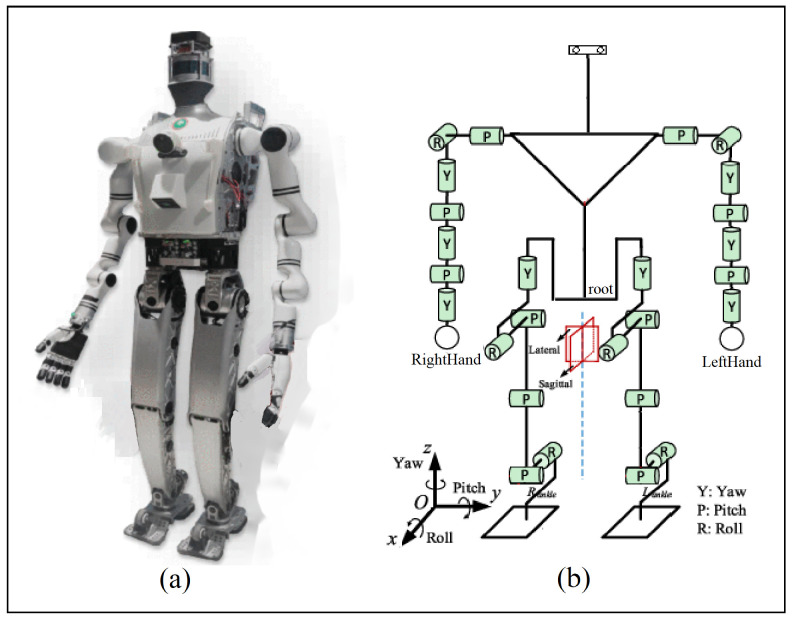
The humanoid robot BHR10. (**a**) Robot platform. (**b**) Kinematic structure.

To verify the actual performance of our proposed method after training, we deploy our proposed method, after training the policies in simulation, to physical hardware. The robot platform in use is BHR10, as is in simulation. The robot is a position-controlled biped robot that weighs 62 kg and stands 1.6 m tall. It is developed by the Beijing Institute of Technology, consisting of a total of 26 DoFs: 6 on both legs and 7 on arms, along with force sensors on both feet, an IMU sensor and an egocentric RGB-D camera. The depth image is further masked such that only objects less than 2.0 m away are kept, discarding the noisy and irrelevant background of the room. The robot platform and its kinematic structure are shown in Figure 9. As of the coordinate system, the $+\hat{x}$ is pointing to the front, $+\hat{y}$ to the left and $+\hat{z}$ up.

### 6.2. Indoor Experiments

**Figure 10:**
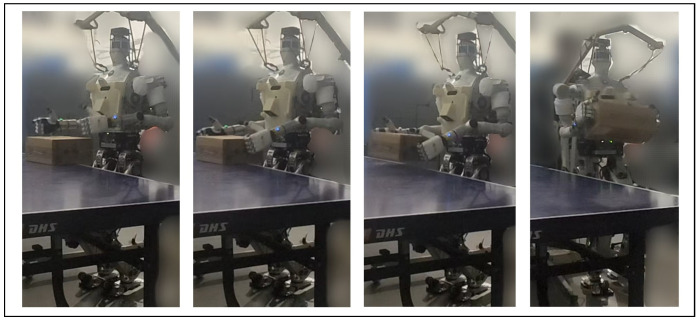
Snapshots of deploying our proposed method on the real BHR10 humanoid robot, performing the load-carrying task. It completed this task successfully 26 out of 30 trial runs.

**Figure 11:**
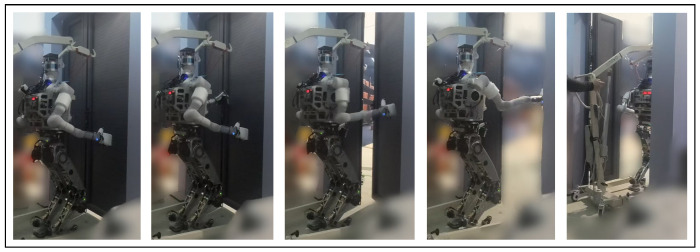
Snapshots of deploying our proposed method on the real BHR10 humanoid robot, performing the door-opening task. It completed this task successfully 24 out of 30 trial runs. The auxiliary door on the left is not part of simulation, and is opened manually in experimental runs to avoid collision with the engine hoist.

We aim to validate our policies’ robustness against different starting positions, sensor inaccuracies, and floating-base dynamics. We test both manipulation tasks described in Section 5. During evaluation, the robot performed each task 30 times in a row. Our proposed method succeeded in 26 out of 30 trials in the Load-carrying task and 24 out of 30 trials in the Door-opening task, respectively. Snapshots of executing these tasks with the real robot platform BHR10 are displayed in Figure 10 and Figure 11, respectively. In the door-opening experiments, the door has an auxiliary part that was not part of previous simulation, and it is opened manually to avoid collision with the engine hoist protecting the robot in case it falls.

### 6.3. Locomotion Stability

**Figure 12:**
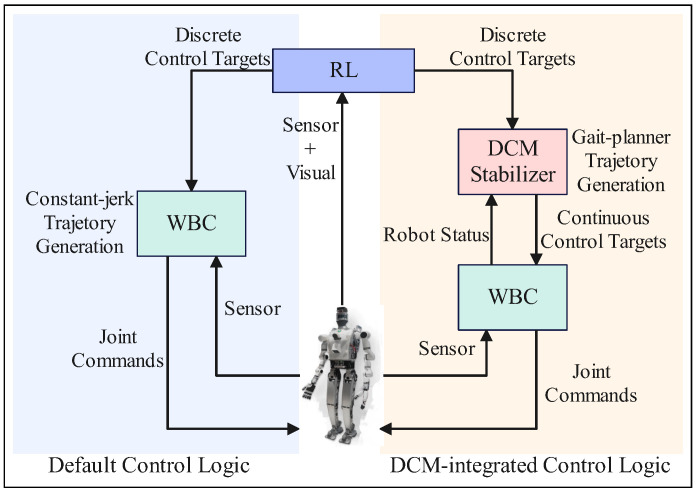
Control logic comparison between the default RL-WBC set-up and the variant with a DCM stabilizer integrated.

Our proposed method implements robot locomotion by directly controlling the target CoM position and feet poses of the robot through WBC. This works in simulation but is too risky in actual experiments. As a result, we replace it with a walking stabilizing controller based on divergent component of motion (DCM) of the linear inverted pendulum model (LIPM) as described in the original work of [51]. It is not difficult to integrate this existing walking controller into our proposed framework, as it is using the same WBC control architecture. In the defautl RL-WBC setup, the WBC layer uses a constant-jerk strategy to interpolate between current value and target value of the control task, which could be risky in bipedal locomotion. In comparison, in the RL-DCM-WBC structure, DCM uses its built-in gait planner to generate continuous reference trajectories (CoM, ZMP and foot poses) according to the discrete target CoM position and feet poses determined by RL; it then utilizes WBC to follow these reference trajectories and reach the same destination state set by RL. A comparison between the control logic of these setups is displayed in Figure 12.

**Figure 13:**
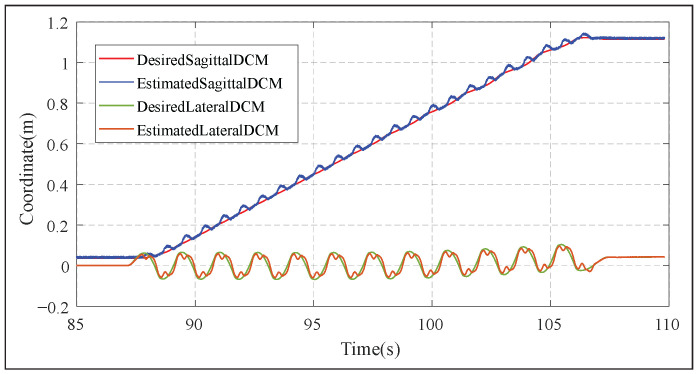
DCM tracking performance while walking pass the door after opening it. Swing leg motions are not accounted for in the walking pattern, driving the DCM away from its reference at each step. This disturbance is compensated by the stabilizer.

Essentially, the DCM-based walking stabilizer is an additionally layer inserted between RL-based motion-planning and WBC-based motion controlling, to boost stability in tasks that involve walking as way of locomotion. This does not reduce the RL policy’s ability to adjust motions, as the DCM stabilizer only executes RL decisions safely. Figure 13 demonstrates the DCM tracking performance of the used walking stabilizer.

**Figure 14:**
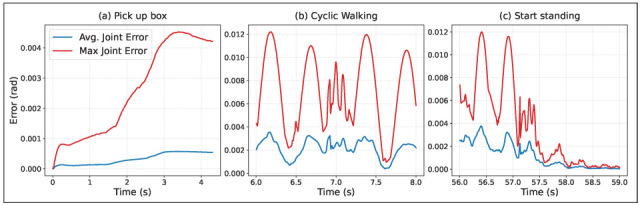
Average and maximum value of absolute joint tracking error during different phases of the box manipulation task: (**a**) Picking-up the box, (**b**) Walking and (**c**) Standing after walking. Two walking cycles are displayed for the cyclic walking phase.

To evaluate control accuracy of the WBC layer and its performance in bridging the sim-to-real gap, we calculate absolute joint tracking errors for all 26 joints at each control interval for the box manipulation task. The result is displayed in Figure 14. As shown in Figure 14, the average joint error is of the order of $10^{-3}\;\mathrm{rad}$ at all times, while the maximum absolute joint error is one order of magnitude greater, capped at approximately 0.01rad. As expected, joint tracking error increases when the robot establishes hand contacts with the box and changes foot contacts during walking.

### 6.4. Manipulation Adjustment

**Figure 15:**
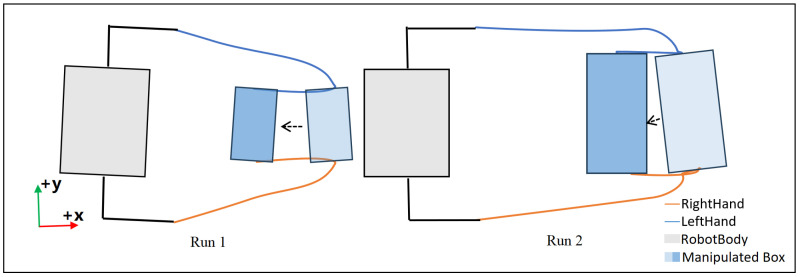
Top-down view of the robot’s end-effectors’ estimated trajectories from standing on ground to picking up different rectangular boxes at different places.

With to the RL layer, our proposed method can adjust the end-effectors’ trajectory by setting different waypoints during manipulation. Figure 15 shows the estimated XY-plane trajectory of the robot’s both hands when it tried to pick up boxes with different sizes and locations in two experimental runs. These trajectories are depicted with simplified depiction of the robot’s and box’s projection in the XY-plane which would facilitate understanding of these trajectories. As shown, the trained policy is capable of adjusting the end-effector trajectories with respect to different sizes and locations of the box. More precisely, in Run 1, the robot’s Left reaches to (0.25m,0.10m) in the robot’s frame to pick up the box, as opposed to (0.33m,−0.19m) in Run 2. This shows that the RL policy has changed the movement target for Left by at least 0.12m to accommodate changes in the environment. After picking up the box and bringing it closer to the robot’s torso, the robot starts the locomotion phase and its manipulation control goal is to keep the box’s relative pose to the robot.

**Figure 16:**
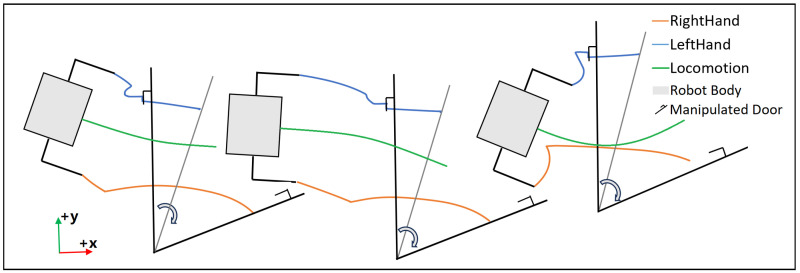
Top-down view of the robot’s end-effectors’ estimated trajectories from standing on ground to manipulating the door handle and door itself in experimental runs with different standing poses.

In a similar fashion, Figure 16 shows the estimated trajectory of the robot’s hands in the X-Y plane when it unlocked the door and then pushed it open with different initial locations and orientations from three experimental runs. Projections of the robot and the door are also displayed. As expected, the policy learns to pushes less if the robot stands further away from the door, as its arm has a limited operation range. After the door has been opened, the policy also learns to adjust its walking trajectory, as the robot starts from different position and orientation, to avoid colliding with door. More precisely, in Run 1, the robot’s RightHand reaches to (0.58m,−0.06m) in the robot’s frame to push the door wide open, as opposed to (0.58m,0.08m) in Run 3. This suggests that the RL policy has changed control target for the RightHand by at least 0.14m due to changes in the robot’s displacement.

Note that in simulation, the door hinge is rather stiff as it only opens up to the angle where the robot’s end-effector stops at. So the robot needs to do another push after moving forward a few steps, as shown in Figure 7. But in the actual experiments, the real door turned out to have much less friction and it continued opening considerably after the robot stopped pushing. So in the actual experiments, the additional push is manually skipped and the robot finished its locomotion non-stop.

## 7. Discussion

In simulation (Figure 6 and Figure 8), our hybrid RL-WBC framework (Method 1) is 2–3 times more successful than the model-based method that cannot adjust motion targets (Method 4). More importantly, our method achieves 83% overall real-world success in different humanoid loco-manipulation tasks. The visuomotor RL policy enables robust responses to object displacement and property changes, adjusting the robot’s end-effectors’ target position by as much as 0.15m in the door-opening experiment (Figure 16) and 0.12m in the box manipulation experiment (Figure 15).

Meanwhile, the model-based motion controller (Equations (1)–(5)) ensures dynamic feasibility under manipulation loads. This framework is facilitated by our training efficiency techniques which accelerated convergence of the RL policy neural network by 50–100%: task-stage prioritized replay focuses learning on near-success transitions, while midway initialization leverages stable interim states to overcome sparse-reward challenges. The simulation and experimental results demonstrate that the hybridization of RL and WBC in the task space is an efficient solution for contact-rich humanoid loco-manipulation tasks. However, there is still 17% failure in real-world experiments, when the robot misses the target, unable to establish proper contact with the box or the door. This could be attributed to increased depth-sensing errors under low-light conditions of the indoor environment.

## 8. Conclusions

The ability to complete loco-manipulation tasks opens up a wide-range of possibilities of applying humanoid robots in real-life scenarios. Current control methods cannot address the problem of varying environment parameters while maintaining versatility. In this paper, we propose a robust and versatile control framework that enables a full-body humanoid robot to perform various loco-manipulation tasks subject to random changes in various initial conditions. It implements a depth-image-based visuomotor RL policy that automatically adjusts targets of the underlying WBC control tasks. Our proposed framework adopts the techniques of mid-way initialization and prioritized experience sampling to facilitate the convergence of the policy network. These techniques are validated in a case study of different loco-manipulation tasks with varying environment parameters. Finally, our proposed method is implemented on a real-world humanoid robot, resulting in an overall 83% success rate in 30 trial runs of box manipulation and door opening tasks, respectively. It can change the robot’s motion targets by more than 0.1cm to adjust to changes in the environment.

Several improvements could be made in the future, such as adding a self-recovery mechanism and enabling the action vector to select a different movement. However, the greatest limitation of our proposed is that it still requires a hand-designed FSM to define the motion sequence. This is inefficient if the humanoid robot is expected to learn a large amount of skills. In the future, we would like to apply learning-from-demonstration methods to compose a sequence of control tasks and targets that can replicate a human motion sequence.

## Figures and Tables

**Table 1:** 6-D pose estimation comparison in simulation.

Architecture	Trans./Rot. Error	Param. Count	Input
Our method	1.8 cm/4.5°	∼1.6M	Depth
ResNet-18	2.6 cm/6.5°	∼12M	RGB
PoseCNN	2.1 cm/5.3°	∼35M	RGB
GDR-Net	1.2 cm/3.1°	∼50M	RGB
FFB6D	0.9 cm/2.5°	∼65M	RGB-D

## Data Availability

Data is available upon request to the corresponding author via email.
